# Editorial: Recent Developments of Deep Learning in Analyzing, Decoding, and Understanding Neuroimaging Signals

**DOI:** 10.3389/fnins.2021.652073

**Published:** 2021-05-21

**Authors:** Junhua Li

**Affiliations:** ^1^Laboratory for Brain-Bionic Intelligence and Computational Neuroscience, Wuyi University, Jiangmen, China; ^2^School of Computer Science and Electronic Engineering, University of Essex, Colchester, United Kingdom

**Keywords:** deep learning, neuroimaging, neurophysiological signals, brain activities classification, brain disease diagnosis

As we know, observation is one of the important ways in which we can understand our surrounding environment and our body itself. In most cases, intermediate measures have to be utilized during observation in order to either have a more precise assessment or obtain a quantitative evaluation. This is vital in the research of the human brain because we are not able to safely probe the brain without measurements, especially for cognitive functions of the brain. Thanks to the advancement of the neuroimaging technique, we can record diverse signals from the brain without any harm to the brain. With these signals, brain activities can be investigated and underlying neural mechanisms can be revealed. To date, diverse neuroimaging techniques have been developed to acquire different signals, such as electroencephalogram (EEG), electrocorticogram (ECoG), and magnetic resonance imaging (MRI). Each type of signal has its own strength and can be utilized together to obtain complementary strengths in some cases. For instance, EEG has a high temporal resolution, which enables us to explore transient changes in the brain. Whereas, it suffers from the drawback of low spatial resolution. This drawback can be relieved by the complimentary signal of functional MRI, which is of high spatial resolution.

Besides the help of diverse signals as mentioned above, another part contributing to the understanding of the brain is machine learning methods. Deep learning, one of the machine learning methods and a prevalent methodology, exhibits success in the fields of image retrieval, speech recognition, and video processing (Lecun et al., 2015). It also shows promising potential in the analyzing, decoding, and understanding of neuroimaging signals (Li et al., 2015; Schirrmeister et al., 2017; Bernard et al., 2018; Goh et al., 2018). Brain activity-related information can be extracted from neuroimaging signals by deep learning and can be represented as more and more abstract and meaningful features through the layers of the deep learning model. Based on the extracted features, brain activities can be understood and brain patterns can be recognized. The research of deep learning in neuroimaging signals sits in the intersection of brain science and machine learning, featuring as an interdisciplinary topic.

To accommodate recent achievements involved in this Research Topic, the paper call was launched in 2018. Based on the peer review and editorial assessment, five papers were finally accepted for publication in this topic. These papers cover three signal modalities (i.e., EEG, ECoG, and MRI) and span over a wide range from brain understanding to clinical applications. A brief introduction to these papers can be found below.

Ghosh et al. utilized a deep learning model called a convolutional neural network (CNN) to discover brain areas and the dominant frequency band of an EEG signal relevant to the exercise of isometric motor grip. According to their study, the deep learning-based method can help refine the frequency band associated with the exercise. A much narrower frequency band (27–29 Hz) was identified, compared to a usually used frequency band of 15–30 Hz. Moreover, the exercise-related brain areas were also identified, which were the contralateral and ipsilateral sensorimotor areas, contralateral prefrontal area, and occipital area. The authors stated that these results were in strong agreement with the inferences drawn in a previous study (Dal Maso et al., 2018). In contrast to this paper, all the others involve patients. The paper written by RaviPrakash et al. states that deep learning makes electrocorticography-based functional mapping (ECoG-FM) comparable to electrocortical stimulation mapping (ESM) in the localization of language regions. Moreover, ECoG-FM is safer to patients and could lower the risk. In their model of deep learning, different deep learning modules such as convolutional network and long short-term memory (LSTM) were included to extract different features. The final decision was made by the majority voting strategy. In this application, deep learning was used to localize the language regions, which facilitated the avoidance of the wrong removal of the language regions during epilepsy surgery. Besides the assistance purposes, deep learning can be utilized to diagnose diseases. Dubreuil-Vall et al. extracted time-frequency features to distinguish the patients with attention deficit hyperactivity disorder (ADHD) from healthy people. Good classification accuracy was achieved by employing the CNN model. They also revealed that the features mainly contributing to the classification were the decreased alpha band power and the increased delta-theta band power around 100 ms for ADHD patients compared to healthy people. Other than the decision at the time, the paper written by Li et al. targets continuous monitoring of the depth of anesthesia (DoA). They proposed a combination of LSTM and a sparse denoising autoencoder to estimate the DoA, and the method achieved a higher performance compared to the other methods shown in the paper. The last paper written by Kuzina et al. does not report a particular clinical application. Instead, the paper focuses on model improvement, proposing knowledge transfer to enhance MRI image segmentation performance in a new dataset by the help of a generative Bayesian prior network.

The studies presented in the papers of this Research Topic target diverse aspects of deep learning in neuroimaging signals, which are interesting and have been part of the progress in recent years. More details of each study can be found by reading respective papers. Finally, I would like to take this opportunity to express my thoughts on future developments. (1) Deep learning models will be more specific to fit with neuroimaging signals by taking signal characteristics and relevant information into consideration. Such an attempt was made in Wang et al. (2020), where kernel size was set to match with brain anatomical structure. (2) A more general deep learning model should be developed to achieve several classification/data analysis tasks, rather than a particular task as now. (3) Accordingly, classification/analysis results should not always be obtained in the last layer of a deep learning model. The results could be generated once there is enough information to do so even before reaching the last layer. For more thoughts beyond deep learning please refer to Li (2020).

## Author Contributions

JL prepared and wrote the manuscript.

## Conflict of Interest

The author declares that the research was conducted in the absence of any commercial or financial relationships that could be construed as a potential conflict of interest.
